# Recent clinical evidence on nutrition, novel pharmacotherapy, and vaccination in inflammatory bowel diseases

**DOI:** 10.3389/fphar.2024.1380878

**Published:** 2024-09-06

**Authors:** Theodora Gheonea, Maria Bogdan, Andreea-Daniela Meca, Ion Rogoveanu, Carmen Oancea

**Affiliations:** ^1^ Center for IBD patients, Faculty of Medicine, University of Medicine and Pharmacy from Craiova, Craiova, Romania; ^2^ Department of Pharmacology, Faculty of Pharmacy, University of Medicine and Pharmacy from Craiova, Craiova, Romania; ^3^ Department of Biochemistry, Faculty of Medicine, University of Medicine and Pharmacy from Craiova, Craiova, Romania

**Keywords:** inflammatory bowel diseases, nutrition, vedolizumab, ustekinumab, risankizumab, mirikizumab, vaccination

## Abstract

Inflammatory bowel diseases (IBD), which enclose Crohn’s disease (CD) and ulcerative colitis (UC), are chronic, relapsing inflammatory ailments. Their specific pathogenesis is not completely clarified, the worldwide incidence and prevalence of IBD has been steadily growing, and there is still not a definitive cure. The management of IBD has become more and more targeted, with specific immune mediators identified to be involved in its pathogenesis. Vedolizumab, a humanised monoclonal antibody binding specifically to the α4β7 integrin, is a gut-selective immunosuppressive biologic drug administered for both CD and UC. With the same indications as vedolizumab, ustekinumab is a fully human IgG1κ monoclonal antibody binding with specificity to the shared p40 protein subunit of human cytokines interleukin (IL)-12 and IL-23. Several selective IL-23p19 monoclonal antibodies (risankizumab, mirikizumab, and guselkumab) have also revealed admirable efficacy and safety in IBD patients. Nutrition is a very important environmental factor associated with the onset and progression of IBD, and the Western diet is considered to contribute to the development of IBD. In this narrative review, our aim is to present an overview of the main results from recent clinical studies on IBD regarding diet, new drug treatments, and also vaccination.

## 1 Introduction

Inflammatory bowel diseases (IBD), regarded as ulcerative colitis (UC) and Crohn’s disease (CD), predispose individuals to long-term progressive intestinal symptoms with unpredictable evolution, that further require lifelong monitoring and treatment (Bhattacharya and Cross, 2020). Patients diagnosed with IBD can experience: intense abdominal pain, potential rectal bleeding, diarrhoea, dehydration, weight loss, anemia, fatigue (Al-Bawardy et al., 2021).

Given the chronic nature of IBD, ongoing medical supervision is essential to monitor disease activity, adjust treatments, and manage potential side effects. Regular consultations with gastroenterologists are pivotal for evaluating treatment efficacy and promptly identifying disease flare-ups, thereby facilitating timely interventions (Lamb et al., 2019).

A multidisciplinary team is essential in managing IBD because it involves various aspects of care: apart from the gastroenterologists who manage the medical treatment, dieticians provide dietary advice to help manage symptoms and nutritional deficiencies. Nurses offer support, education, and assistance with medication management, psychologists or counselors help address the emotional and mental health aspects, including coping strategies and stress management. Surgeons may be involved if surgical intervention is necessary. Pharmacists ensure proper medication management and patient education regarding drug interactions and side effects. This team approach ensures comprehensive care, addressing all facets of the patient’s health and wellbeing (Lamb et al., 2019).

Quality of life considerations for IBD individuals span physical, emotional, and social dimensions. Effective symptom management through pharmacological interventions and lifestyle adaptations, coupled with addressing nutritional deficiencies, significantly contributes to overall wellbeing (Lamb et al., 2019).

Standard non-biological therapies have been administered for decades, although neither thiopurines (azathioprine, mercaptopurine), corticosteroids (prednisone, budesonide, methylprednisolone, hydrocortisone) nor amino-salicylates (mesalazine, sulfasalazine, 5-aminosalicylate) have proven general efficacy or long-term safety (Bhattacharya and Cross, 2020). Nevertheless, the approved treatments in IBD have not influenced pathological evolution (Al-Bawardy et al., 2021). Patients with IBD are also predisposed to episodes of symptoms exacerbation or disease remission even under treatment, characterising therefore these diseases as unpredictable and difficult to manage (Lamb et al., 2019). Novel medications for IBD, that reduced the need of hospitalization or surgical intervention while leading to quality life improvement, include biological agents such as.

- anti-TNF-α agents (infliximab, certolizumab, adalimumab) in patients diagnosed with moderate-to-severe IBD or in non-responders to conventional therapy (Feuerstein et al., 2020);
- anti-α4β7 integrin monoclonal antibody (vedolizumab) approved as option in individuals who are not responsive to conventional anti-TNF-α medication and with lower incidence of leukoencephalopathy compared to natalizumab (the first approved humanized anti-adhesion monoclonal antibody in IBD) (Vermeire et al., 2022);
- anti-IL-12/IL-23 monoclonal antibody (ustekinumab), with a more important clinical response after long-term treatment and with the advantage of intravenously or subcutaneously administration (Hanžel and D'Haens, 2020);
- small molecules that act as JAK inhibitors (tofacitinib), with major benefits of low cost and oral administration in comparison with other biologic drugs (Sandborn et al., 2017).

In Romania, a step-up therapy is currently recommended in IBD patients, which further includes: standard monotherapy (5-aminosalycilates derivates, corticosteroids or immunomodulators including methotrexate), followed by associated standard therapy, or biologics (infliximab, adalimumab, vedolizumab or ustekinumab) in case of moderate-severe manifestations or non-responders to initial agents (Romanian National Health House of Inssurances, 2017; Romanian National Health House of Insurrances, 2021; Romanian National Health House of Insurrances, 2023). Vedolizumab and ustekinumab have been approved recently in treatment protocols and are recommended only in cases of insufficient or no response after anti-TNF-α agents (Romanian National Health House of Inssurances, 2017; Romanian National Health House of Insurrances, 2021; Romanian National Health House of Insurrances, 2023). On the other hand, biologic agents lead to immunosupression and may complicate vaccine administration in patients diagnosed with IBD (GBD, 2017 Inflammatory Bowel Diseases Collaborators, 2020).

The purpose of our review is to bring a better and updated understanding towards the activated pathological mechanisms in IBD patients during nutrition, novel pharmacotherapy (vedolizumab, ustekinumab, anti-IL23 p19 drugs), and vaccination.

## 2 Nutrition and IBD

The global incidence and prevalence of IBD continue to rise, with over 6.8 million individuals diagnosed worldwide in 2017, and three million cases in the United States alone (GBD, 2017 Inflammatory Bowel Diseases Collaborators, 2020; Centers for Disease Control and Prevention, 2023). IBD also represent an economical burden around the globe, as part of the top five most expensive gastroenterological pathologies (GBD, 2017 Inflammatory Bowel Diseases Collaborators, 2020). A rapid rise of IBD has also been noted in Europe in the last years, increasing the necessity to monitor and to guide both patients regarding nutritional intervention and physician regarding pharmacotherapeutic and vaccination management (GBD, 2017 Inflammatory Bowel Diseases Collaborators, 2020). Moreover, this chronic condition has no clear etiology and none of the already approved treatments is curative, nor is able to fully prevent relapsing intestinal inflammation (Lamb et al., 2019; Roncoroni et al., 2022). The etiology and pathogenesis of IBD are presently recognized as an intricate interaction involving immunological, microbiological, genetic, and environmental factors, with imbalance in cytokines production (such as TNF-α, IL-6, IL-10, IL-17, IL-22) and other signalling pathways (Al-Bawardy et al., 2021; Lamb et al., 2019). Another factor involved in IBD progression is the poor quality of food or inadequate nutritional behavior, although it is still unclear how selective nutritional components affect intestinal barriers and pathological mechanisms (Roncoroni et al., 2022; Godala et al., 2022). The frequent disease relapses, the psycho-social implications and adverse effects of long-term therapy can influence the patients’ adherence to treatment and therefore, standardised treatment guidelines together with nutritional interventions could better support pharmacological management (Roncoroni et al., 2022; Godala et al., 2022). However, evidence on how nutritional interventions or long-term treatment can affect IBD progression is still poor (Bhattacharya and Cross, 2020; Roncoroni et al., 2022).

Nutrition stands as a pivotal environmental factor linked to the initiation and advancement of IBD. The current body of evidence suggests the existence of an intestinal dysbiosis accompanied by an abnormal immune reaction in individuals with genetic susceptibility, likely influenced by alterations in environmental factors such as diet (Campmans-Kuijpers and Dijkstra, 2021). Given the highest prevalence of IBD in Western countries, it is hypothesized that the Western dietary pattern - characterized by high fat and sugar intake and low consumption of vegetables and fruits - plays a contributory role in IBD development. Additionally, it is widely acknowledged that the Western diet diminishes the diversity of the gut microbiome (Campmans-Kuijpers and Dijkstra, 2021).

Research from Asia, Latin America, and Africa highlights a rising incidence and prevalence of IBD, reflecting shifts towards more Westernized lifestyles characterized by increased urbanization and dietary changes (GBD, 2017 Inflammatory Bowel Diseases Collaborators, 2020; Centers for Disease Control and Prevention, 2023). A rise in age-standardized prevalence rates of IBD in regions historically characterized by low incidence (such as East and South Asia, Oceania, and sub-Saharan Africa), is observed (GBD, 2017 Inflammatory Bowel Diseases Collaborators, 2020; Centers for Disease Control and Prevention, 2023). This increase is likely influenced by several factors, including advancements in the socioeconomic status of newly industrialized nations, shifts in dietary habits and lifestyles, enhanced sanitation practices, alterations in gut microbiota, and environmental changes. These factors collectively contribute to an elevated risk of developing IBD in these regions (GBD, 2017 Inflammatory Bowel Diseases Collaborators, 2020; Centers for Disease Control and Prevention, 2023). In Asia, urban populations are particularly affected, with changes in diet and environmental factors contributing to the observed increase. Previous case-control studies conducted before the onset of illness, including those in Japan, have indicated higher consumption of sugar, fast foods, chocolate, and cola drinks among IBD individuals, as well as a reduction in dietary fiber from fruits and vegetables specifically noted in cases of CD (Chiba et al., 2010).

Due to the significant influence of nutrition on the pathophysiology of IBD, various dietary approaches have been investigated to determine optimal food choices. Chiba et al. illustrated that a semi-vegetarian diet exhibited greater efficacy in sustaining remission among CD patients compared to an omnivorous diet (Chiba et al., 2010).

**FIGURE 1:**
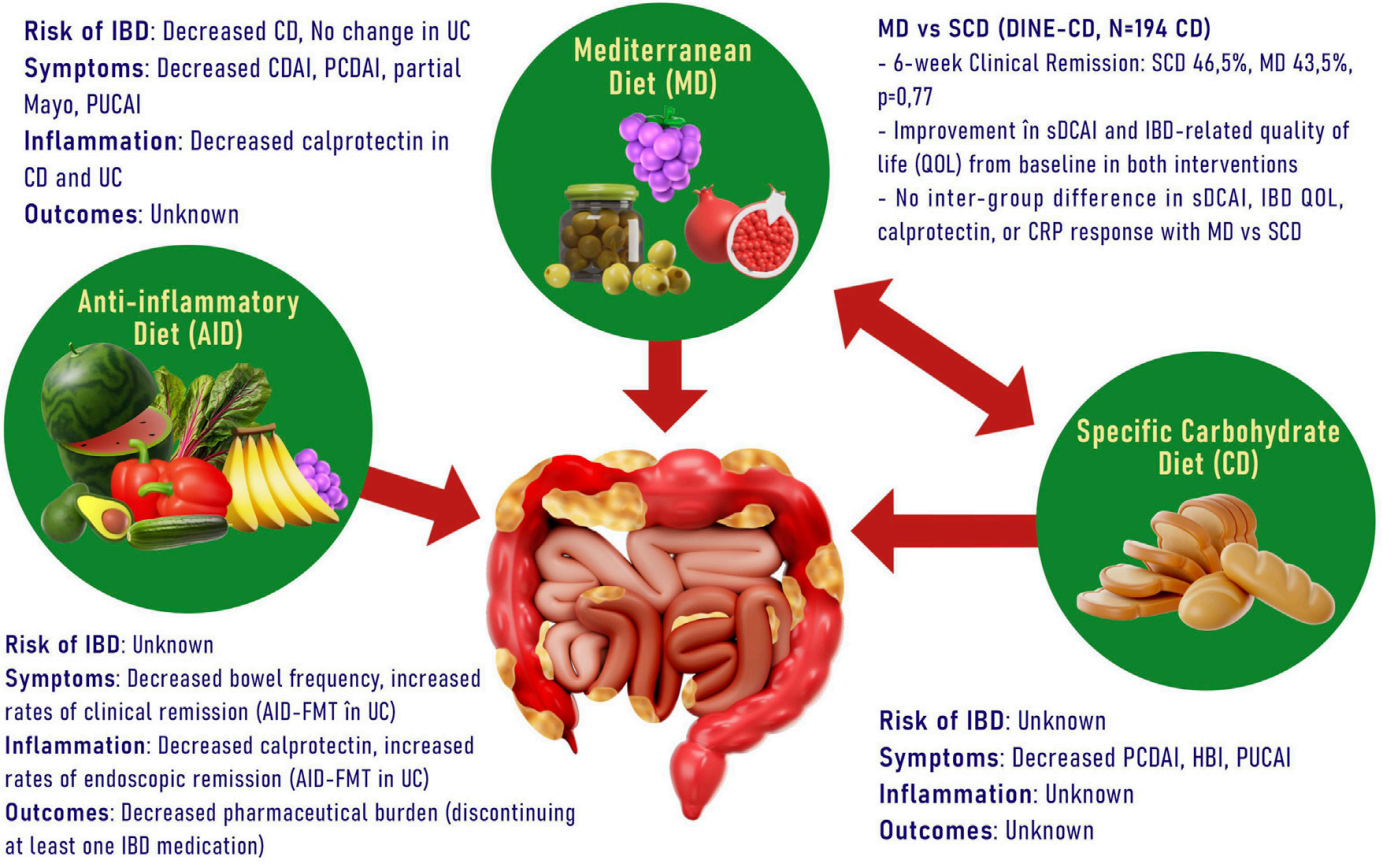
Different types of diets and their benefits in IBD. (CD, Crohn Disease; CDAI, Crohn Disease Activity Index; PCDAI, Pediatric Crohn Disease Activity Index; PUCAI, Pediatric Ulcerative Colitis Activity Index; sDCAI, Distribution, Chronicity, Activity in IBD; CRP, C reactive protein; FMT, Fecal Microbiota Transplantation; HBI, Harvey-Bradshaw Index).

The Anti-Inflammatory Diet for IBD (IBD-AID) is a dietary regimen designed to reduce inflammation, which involves limiting specific carbohydrates, incorporating pre- and probiotic foods, and adjusting the composition of dietary fatty acids (Chiba et al., 2010). This dietary approach has demonstrated some efficacy as adjunctive therapy in the treatment of patients with IBD. Furthermore, specific carbohydrate diets, initially intended for the management of celiac disease in children and involving the elimination of all grain varieties, have been explored for their potential to alter the microbiome in individuals with IBD (Suskind et al., 2014). Recently, a strategy involving partial enteral nutrition alongside a diet excluding certain carbohydrates (CD exclusion diet) has demonstrated utility in both pediatric and adult patients who have not responded to biologic therapy (Figure 1).

Recent findings have highlighted the impact of contemporary urban diets on IBD. In a prospective cohort spanning 21 countries and encompassing 116,087 individuals, the consumption of ultra-processed foods was linked to the onset of IBD in a dose-dependent fashion, with a notable increase observed in individuals consuming five or more servings per day compared to those consuming fewer than one serving per day (Narula et al., 2021).

The IOIBD provides comprehensive dietary guidelines for all food groups when no specific dietary indications are present. While a moderate to high intake of fruits and vegetables is recommended for CD patients, those with intestinal strictures should be cautious about insoluble fiber from sources like spinach, tomatoes, raspberries, kiwi, and avocado (Chiba et al., 2010). Complex carbohydrates and simple sugars should follow general health standards, with a low FODMAP diet suggested for CD and UC patients with functional bowel disorders. There is no need to restrict wheat protein or gluten intake for IBD patients (Chiba et al., 2010).

For protein sources, there are no restrictions on red meat, poultry, or eggs for CD patients, but UC patients should moderate red meat intake. All IBD patients are advised to avoid trans fats and saturated fats, and UC patients should reduce myristic acid from sources like palm oil, coconut oil, beef, and dairy, while increasing omega-3 fatty acids (Chiba et al., 2010). No specific recommendations exist for pasteurized dairy products, but all IBD patients should avoid processed foods with additives like maltodextrin, emulsifiers, thickeners, nanoparticles, and sulfated compounds (Chiba et al., 2010).

Maintaining a long-term food journal and symptoms journal is crucial. Consistently tracking daily food intake helps identify potential triggers, while documenting symptoms over time aids in monitoring disease progression and response to treatment. This ongoing task is very important, as it facilitates better communication with healthcare providers and contributes to more personalized and effective disease management.

In a prospective study involving 412 individuals with ulcerative colitis (UC) in remission, who received treatment with an aminosalicylate, researchers found that higher dietary intake of myristic acid, a saturated fatty acid present in nutmeg, coconut oil, and cow’s milk, was linked to an increased risk of disease relapse (Campmans-Kuijpers and Dijkstra, 2021). Moreover, processed and unprocessed meats, containing elevated levels of organic sulfur and sulfate additives, may lead to higher sulfate levels, promoting the production of hydrogen sulfide by gut microbes. The resultant end products of protein fermentation, particularly hydrogen sulfide (H2S), ammonia, and to a lesser extent, phenols, were observed to have adverse effects on the colonic microenvironment and epithelial health (Campmans-Kuijpers and Dijkstra, 2021).

Obesity present among people suffering from IBD led to a lower prevalence of remission, but with higher scores of central adverse reactions (such as anxiety, lethargy, depression), pain and social functionality inferior to PROMIS (Patient-Reported Outcomes Measurement questionnaire scores Information System), compared to non-obese patients with IBD (Jain et al., 2019).

In a cohort study comprising 7296 individuals diagnosed with IBD (4,748 adults with CD, among whom 19.5% were obese and 2,548 patients with UC, with 20.3% being obese), Jain et al. noted an independent association between obesity and a heightened risk of persistent disease activity or relapse in patients with CD (Jain et al., 2019). In a separate cohort study, encompassing a significantly larger population of 42,285 individuals diagnosed with IBD (of whom 12.4% were obese), Nguyen and collaborators found that obese patients with this condition experienced a greater annual burden and incurred higher hospitalization costs compared to their non-obese counterparts (Nguyen et al., 2019).

Data from the current literature show that herbal supplements, dietary minerals and vitamins and exercise have attracted special attention for their anti-inflammatory activity and possible utility in the treatment of IBD (Torres et al., 2019; Cheifetz et al., 2017).

Therefore, preclinical results from experimental studies in mice indicated that cannabinoid receptor activation facilitates protective mechanisms in experimentally induced colitis. Consistent with these observations, two clinical trials documented that cannabis was effective in alleviating symptoms of IBD (Oligschlaeger et al., 2019), indicating its potential ability to treat IBD. However, further investigations are needed to know if cannabis is really able to positively influence the course of the disease.

Given the significant impact of stress on both the structural and functional characteristics of the microbiome, numerous studies have delved into the potential of psychobiotics in managing stress-related conditions. Psychobiotics encompass probiotics or prebiotics capable of modulating the commensal gut microbiota. When consumed in sufficient quantities, they can indirectly yield beneficial psychiatric effects in cases of psychopathology (Sarkar et al., 2016). Extensively reviewed evidence indicates that in both experimentally induced colitis and IBD diagnosed in humans, pre- and probiotics have shown favorable outcomes in prevention (Sarkar et al., 2016; Akram et al., 2019). They achieve this by modulating the trophic functions of the microbiota, enhancing the integrity of the gut mucosal barrier, and facilitating anti-inflammatory responses (Akram et al., 2019). It is plausible to consider psychobiotics as therapeutic agents for modulating the gut-microbiota axis, potentially enhancing psychological functions within the context of IBD. This assumption is supported by evidence suggesting that the consumption of psychobiotics may confer antidepressant effects, manifesting as improvements in mood and reductions in urinary free cortisol levels (Sarkar et al., 2016).

## 3 Vedolizumab

Vedolizumab is a humanized monoclonal antibody engineered to alleviate intestinal inflammation by interfering with cell trafficking to the intestine. This is achieved by specifically targeting the α4β7 heterodimer (Feagan et al., 2013).

The α4β7 receptor, a transmembrane integrin, is expressed on the majority of leukocytes and plays a pivotal role in facilitating their migration to intestinal-associated lymphoid tissues. This migration is mediated through interaction with its main ligand, mucosal addressin cell-expressed adhesion molecule 1 (MadCAM-1), which is typically found in gut-associated lymphoid areas (Ohmatsu et al., 2010). MAdCAM-1 can influence recirculation of memory T cells through mucosal tissues and is upregulated during inflammation (Briskin et al., 1997).

While vedolizumab has been approved by the FDA for the treatment of UC and CD since 2014, the specific molecular mechanisms underlying its action in humans have not yet been fully elucidated and remain subject to further investigation (Pang et al., 2023; Luzentales-Simpson et al., 2021). In contrast to natalizumab, vedolizumab does not disrupt lymphocyte trafficking to brain tissue (Feagan et al., 2013).

VISIBLE one was a phase 3, double-blind study conducted at 141 sites across 29 countries from 18 December 2015, to 21 August 2018. Patients diagnosed with moderately to severely active UC were treated with vedolizumab administered intravenously at a dose of 300 mg at weeks 0 and 2. Following clinical response at week 6, patients were randomly allocated to maintenance therapy with subcutaneous vedolizumab at a dose of 108 mg every 2 weeks, intravenous vedolizumab at a dose of 300 mg every 8 weeks, or placebo. The primary endpoint was clinical remission at week 52, defined as a total Mayo score of ≤2 with no subscore exceeding 1 (Sandborn et al., 2020).

Out of the 216 randomized patients, 46.2% in the subcutaneous vedolizumab group, 42.6% in the intravenous vedolizumab group, and 14.3% in the placebo group achieved clinical remission at week 52. Additionally, the subcutaneous vedolizumab group exhibited greater endoscopic improvement and sustained clinical response at week 52 compared to the placebo group. The safety profiles of subcutaneous and intravenous vedolizumab were comparable (Sandborn et al., 2020).

GEMINI LTS is an open-label phase 3, single-arm study conducted in various countries among patients diagnosed with moderately to severely active UC or CD. It comprised participants from a long-term phase 2 study and three phase 3 trials, encompassing a cohort of both vedolizumab-naive (*de novo*) patients with UC or CD (Loftus et al., 2020). GEMINI LTS stands as the most extensive and prolonged clinical trial conducted thus far to evaluate the advantages and drawbacks of prolonged vedolizumab therapy in individuals diagnosed with moderately to severely active UC or CD. This final report offers a comprehensive analysis of safety over a median cumulative exposure to vedolizumab spanning 42.4 months (range: 0.03–112.2 months) for UC patients and 31.5 months (range: 0.03–100. months) for patients with CD (Loftus et al., 2017; Vermeire et al., 2017).

Long-term treatment with vedolizumab consistently upheld or enhanced health-related quality of life (HRQOL) in individuals diagnosed with moderate to severely active UC or CD. The array of validated assessment tools utilized to showcase improvements in HRQOL within the GEMINI LTS trial offers additional evidence of the advantages of prolonged vedolizumab therapy on both disease-specific and global indicators of patient wellbeing. Moreover, GEMINI LTS revealed the enduring effectiveness of continuous vedolizumab treatment in patients with IBD, with some individuals experiencing benefits for up to 9 years (Loftus et al., 2020).

The VARSITY study, conducted in 2021, sought to compare the effectiveness and safety of vedolizumab administered intravenously with adalimumab administered subcutaneously in a cohort of 769 participants diagnosed with UC (Peyrin-Biroulet et al., 2021). At baseline, the mean histologic disease activity was comparable between the groups receiving vedolizumab and adalimumab. Vedolizumab demonstrated superior histological remission compared to adalimumab at week 14. Furthermore, at week 52, vedolizumab exhibited higher rates of mucosal healing (defined as a composite endpoint of histological plus endoscopic improvement) compared to adalimumab (Peyrin-Biroulet et al., 2021).

In another study conducted in 2022 by Onali and colleagues, involving a total of 470 patients diagnosed with CD (239 individuals who received ustekinumab and 231 who received vedolizumab), significant differences were observed at week 52. The rates of clinical remission were higher in patients treated with vedolizumab compared to ustekinumab (55.5% vs. 42.5*%, p* = 0.01), as were the rates of steroid-free remission (51.1% vs. 40.6*%, p* = 0.038). However, at week 52, objective response and remission rates presented high percentages of similarity between the two treatment groups. Clinical response at week 26 was predictive of steroid-free remission at week 52 in both categories of patients. Moreover, safety profiles were comparable between the two groups, with a higher rate of clinical remission in individuals who administered vedolizumab compared to the ones who administered ustekinumab at 1 year (Onali et al., 2022).

The VISIBLE two study is a phase 3 trial, randomized, double-blind, and placebo-controlled, evaluating subcutaneous vedolizumab as maintenance therapy in adults with moderately to severely active CD. At week 6, 50.6% of randomized patients achieved clinical remission, and 84.4% showed improved clinical response. By week 52, a significantly higher proportion of patients receiving subcutaneous vedolizumab (48.0%) compared to placebo (34.3%) were in clinical remission. Similarly, improved clinical response at week 52 was observed in 52.0% and 44.8% of patients receiving subcutaneous vedolizumab versus placebo, respectively. While statistical significance for steroid-free clinical remission at week 52 was not reached due to the lack of significance for improved clinical response, the rates were 45.3% in the subcutaneous vedolizumab arm versus 18.2% in the placebo arm. Among patients previously treated with anti-TNF, 48.6% versus 42.9% in the vedolizumab subcutaneously and placebo arms, respectively, achieved clinical remission at week 52 (Vermeire et al., 2022).

Vedolizumab exerts its effect by impeding the migration of circulating immune cells to the mucosa and exhibits selectivity for the gut by interacting with mucosal adhesion molecules. This selective mechanism of action can confer a distinct advantage in terms of long-term safety (Vermeire et al., 2019) and may also account for the longer duration required to induce remission (typically 12–16 weeks) compared to TNF inhibitors. Hence, vedolizumab may not be the optimal choice for patients with severe BC featuring acute involvement, as it necessitates a swift response to treatment (Sulz et al., 2020). It is essential to acknowledge that vedolizumab does not exert effects on the extraintestinal manifestations of IBD (Sulz et al., 2020).

### 3.1 Vedolizumab and nutrition

Recent studies have proposed the Nutritional Risk Index (NRI), Control of Nutritional Status (CONUT), and Universal Malnutrition Screening Tool (MUST) as potential prognostic factors for UC activity. A pilot study was conducted to verify the hypothesis that NRI, CONUT, and MUST could serve as cost-effective and efficient predictive biomarkers of response in UC patients undergoing treatment with vedolizumab (Sobolewska-Włodarczyk et al., 2023).

In the study, 21 men and 11 women underwent 52 weeks of vedolizumab therapy. Nutritional status indicators, including NRI, CONUT, and MUST scores, were assessed for each UC patient at the initiation of biologic treatment. The findings revealed that in this cohort, the MUST score was notably lower in UC patients who achieved clinical remission at week 14 during induction therapy with vedolizumab (0.33 ± 0.49 vs. 1.37 ± 0.83; *p* = 0.002). Additionally, analysis indicated lower baseline NRI and CONUT scores in patients experiencing clinical remission at week 14 (NRI: 96.42 ± 4.29 vs. 101.41 ± 7.09; *p* = 0.024; CONUT: 1.00 ± 1.08 vs. 2.16 ± 1.08; *p* = 0.04). Consequently, nutritional indicators (such as NRI, MUST, and CONUT) for individuals’ status may serve as valuable predictors for disease remission at week 14 after vedolizumab pharmacotherapy in UC patients. Furthermore, the study suggests that nutritional intervention combined with vedolizumab treatment could be a beneficial approach for attaining remission (Sobolewska-Włodarczyk et al., 2023).

## 4 Ustekinumab

Ustekinumab acts by binding to the p40 subunit that is shared by both interleukin-12 (IL-12) and interleukin-23 (IL-23), thereby inhibiting their interaction with the IL-12 receptor β1 subunit within the IL-12 and IL-23 receptor complexes (Benson et al., 2011).

The IM-UNITI study investigated the effectiveness and safety of ustekinumab treatment over a 5-year period in patients with CD. Individuals who completed safety and efficacy evaluations at week 44 of the maintenance study were eligible to continue therapy (Sandborn et al., 2022a). Unblinding occurred following completion of the maintenance study analyses in August 2015, and placebo-treated patients were discontinued from the study after disclosure. Efficacy assessments were conducted from 12 to 12 weeks since baseline and also at subsequent dosing visits until week 252. Serum concentrations of ustekinumab and anti-drug antibodies were measured at weeks 252 and 272, respectively (Sandborn et al., 2022a). At week 252, 34.4% of patients receiving ustekinumab treatment every 8 weeks and 28.7% of those receiving treatment every 12 weeks achieved clinical remission. Among patients continuing into the long-term treatment extension, remission rates were 54.9% and 45.2% respectively. Ustekinumab serum concentrations remained consistent throughout the extended long-term treatment. Anti-drug antibodies were present in 5.8% of ustekinumab-treated patients during induction and maintenance phases, persisting into the long-term extension (Sandborn et al., 2022a).

The objective of the UNIFI study was to assess the effectiveness and safety of maintaining ustekinumab treatment for a duration of 3 years in patients with UC. Among patients randomized to the ustekinumab every 12 weeks and every 8 weeks groups at baseline maintenance, 54.1% and 56.3%, respectively, achieved symptomatic remission at week 152 (Abreu et al., 2022). In total, 20% of patients discontinued the treatment with ustekinumab, with a discontinuation rate of 10% among ustekinumab-naïve patients and 30% among biologic-exposed patients. Among patients who achieved symptomatic remission at year 3, 94.6% and 98.0% were also without corticosteroid treatment, respectively. Corticosteroid-free symptomatic remission rates in the ustekinumab every 12 weeks and every 8 weeks groups were 51.2% and 55.1%, respectively, at week 152. Notably, remission rates were higher for biologic-naïve patients compared to those with a history of biological insufficiency (Abreu et al., 2022).

In 2021, a multicenter cohort study was conducted involving 108 patients with UC who received ustekinumab treatment. Among them, 56.5% were women, 91.7% had prior exposure to anti-TNF, 39.8% had more than two previous biological exposures, and 57.4% were receiving oral corticosteroid therapy. Nearly 40% of UC patients in the study achieved remission following ustekinumab induction. However, over 40% required ustekinumab therapy intensification, which was notably associated with having more than two prior biologic exposures. Among patients who underwent dose escalation, over 50% achieved corticosteroid-free remission, although those with minimal or no response to induction were less likely to achieve remission after intensification (Dalal et al., 2022).

### 4.1 Ustekinumab and nutrition

An article published in 2021 documents the case of a 10-year-old girl diagnosed with CD. Alongside treatment with 5-aminosalicylic acid, transient prednisolone, and ustekinumab, she was prescribed a low-fat diet. By week 75, the patient achieved clinical remission that could also be visualized through endoscopy, as evidenced by the weighted pediatric CD activity index, fecal calprotectin levels, and colonoscopy results. Notably, she did not experience adverse events such as infusion reactions or increased susceptibility to infections during this period. These findings suggest that ustekinumab, when used as the initial biological agent, can be an effective and safe treatment for CD in pediatric patients, with diet playing a potentially important role as an adjunctive factor (Fujita et al., 2021).

## 5 Anti-IL23 p19 drugs

Heterodimeric cytokines IL-12, IL-23, IL-27 and IL-35 are part of the IL-12 group (Parigi et al., 2022). IL23 contains a specific p19 subunit and a shared p40 subunit, and is mainly produced by macrophages, monocytes, and activated dendritic cells after Toll-like receptor signalling (Noviello et al., 2021). Due to its activity on both innate and adaptive immune pathways, IL-23 has been involved in multiple autoimmune pathologic processes (Gottlieb and Sands, 2022). Activating various target cells, IL23 is able to determine a strong proinflammatory reaction (Noviello et al., 2021) and has an essential role in the pathogenesis of UC and CD, promoting a Th17 cell-related immune response (Parigi et al., 2022; Bretto et al., 2023).

Different studies have proved that IBD patients have higher serum, plasma, and intestinal levels of both IL-23 and Th17 cytokines. Furthermore, the IL-23 receptor affected or loss of functionality and IL-23 inhibition have been investigated by various researchers, who have further shown connection with diminished risk of IBD (Gottlieb and Sands, 2022). Risankizumab, mirikizumab, and guselkumab are three monoclonal antibodies currently approved or in advanced clinical trials for either CD or UC.

Risankizumab is a humanized IgG1 monoclonal antibody that is able to target the p19 subunit on the IL-23 (Horst and Cross, 2023; Sakkas et al., 2019) and has proven efficacy in psoriasis, psoriatic arthritis and CD (Baeten et al., 2018). This compound represents the first specific IL-23 inhibitor as approved therapy for moderately to severely active forms of CD in adult individuals who experienced an inadequate response to or were not tolerant to conventional or a biologic pharmacotherapy, in the European Union, USA, and Canada in 2022 (Dubinsky M. et al., 2023; European Medicines Agency, 2023a).

Risankizumab is available as a single use vial containing 600 mg of risankizumab in 10.0 mL of solution. It must be diluted before being administered by a healthcare professional as an intravenous infusion while using aseptic technique. As the recommended risankizumab regimen has two parts, for the subsequent subcutaneous dosing regimen it is available as a single-use 1-mL pre-filled pen/syringe. The safety profile observed in CD patients treated with risankizumab was in accordance with the one observed in individuals diagnosed with psoriasis. The risk of infection may rise due to risankizumab therapy, and upper respiratory infections (13% in psoriasis, 15.6% in CD) were registered as the most frequently adverse reactions (European Medicines Agency, 2023a).

In a review article from 2023, Clement et al. concluded that in the elderly, risankizumab has favorable side effect profiles with regards to malignancy and infections and can be considered a first line treatment option (Clement et al., 2023).

Mirikizumab is another monoclonal antibody formulated as a humanised immunoglobulin G4 (IgG4)-variant. Mirikizumab is able to bind to the p19 subunit of IL-23, clinically efficient and with a favourable safety profile in psoriasis, UC and CD (Dubinsky MC. et al., 2023; Steere et al., 2023).

It showed clinical advantage, also for bowel urgency, in two phase 3 trials (LUCENT-1 and LUCENT-2) in individuals with moderately to severely active forms of UC, which comprised 52 weeks of continuous therapy. Nevertheless, In LUCENT-1, patients randomly received blinded mirikizumab 300 mg I.V. every 4 weeks, while in LUCENT-2, patients were re-randomized to mirikizumab 200 mg S.C. or placebo S.C. (Dubinsky et al., 2022a).

In a randomised clinical trial in individuals with moderate-to active forms of UC, a phase 2 study, with a duration of 52-week, mirikizumab proved to be efficacious and well-tolerated, and meaningfully diminished stool frequency, bowel urgency, and rectal bleeding (Dubinsky et al., 2022b).

In 2023, mirikizumab was approved in Japan (Keam, 2023) and in the European Union for the adult individuals’ treatment with moderate-severe active UC who were intolerant to or have not had an adequate response or even lost response to either conventional or a biologic therapy (European Medicines Agency, 2023b).

Similar to risankizumab, the recommended regimen of mirikizumab is divided in two sections. For the induction dose, a single use vial containing 300 mg of mirikizumab in 15 mL of solution should be administered by intravenous infusion, after dilution. For the maintenance dose, it is available as a single-use 1-mL pre-filled pen/syringe for subcutaneous administration. Upper respiratory tract infections (7.9%, most often nasopharyngitis), various injection site reactions (during the maintenance period), rash and headache were noted as the most often adverse reactions. Like risankizumab, mirikizumab is contraindicated in clinically important active infections (active tuberculosis) (European Medicines Agency, 2023b).

Guselkumab is approved for treatment in patients diagnosed with moderate to severe plaque psoriasis and with active psoriatic arthritis in several countries (Peyrin-Biroulet et al., 2023). It represents the first monoclonal antibody fully designed as human immunoglobulin G1 lambda (IgG1 λ) selectively aiming and coupling to IL-23; its blocking action is caused by the interaction with the receptor positioned at the cellular surface that usually is bound by the IL23 p19 subunit, further inhibiting signalling pathwats specifically mediated through IL-23.

Guselkumab can be administered intravenously or subcutaneously (Bretto et al., 2023). Guselkumab is packed as a mono-dose single-use 1-mL prefilled syringe, and has no absolute contraindications and no negative interactions with gastrointestinal system. With a major safety profile, after healthcare professionals’ training, it can be also administered by patients themselves (Valenti et al., 2022).

In two recent placebo-controlled trials included in phase 2 and double-blinded, the safety and efficacy of guselkumab were observed and analyzed, in individuals with either moderately to severely active CD (“GALAXI-1 study”) or moderately to severely active UC (“QUASAR Phase 2b Induction Study”) with prior intolerance and/or inadequate response to corticosteroids, immunosuppressants, or advanced therapy. Patients were randomly treated with guselkumab 200 mg, 600 mg, or 1,200 mg I.V. (in “GALAXI-1 study”), and 200 or 400 mg (in “QUASAR Phase 2b Induction Study”) or placebo at weeks 0, 4, and 8. In both studies, guselkumab therapy demonstrated superior efficacy versus placebo, and efficacy and safety were similar between guselkumab dose groups at week 12 (Peyrin-Biroulet et al., 2023; Sandborn et al., 2022b).

### 5.1 Anti-IL23 p19 drugs and nutrition

In recent times, there has been a surge in analyses focusing on the gut microbiome and metabolome, with numerous promising studies dedicated to restoring a healthy balance in the gut microbiome. These advancements offer new hope for individuals affected by IBD (Elhag et al., 2022). In this field, risankizumab has been studied in moderate-to-severe CD in a phase II randomized, placebo-controlled trial (Parigi et al., 2022). This molecule binds to interleukin 23 to prevent the cytokine binding to its receptor and therefore decrease inflammation (Risankizumab for psoriasis, 2020). This process can be influenced to help these molecules through nutritional interventions. Targeting inflammatory pathways through nutritional interventions could potentially offer a sustainable approach to treatment strategies for chronic immune-mediated diseases. The nutritional curcumin (CUR), that can be found in turmeric, is beneficial in cell-mediated autoimmune diseases. CUR silences IL-23/Th17-mediated pathology by enhancing HO-1/STAT3 interaction in CD (Brück et al., 2017). There is also a study made on turmeric-derived nanovesicles (TNV) or alleviation of colitis. Oral administration of TNVs demonstrated outstanding anti-inflammatory efficacy by restoring the compromised intestinal barrier, modulating the gut microbiota, and altering the macrophage phenotype (Gao et al., 2022).

Individuals with CD were observed to have an elevated population of innate lymphocytes expressing RORt+ in the lamina propria compared to controls. This increase was found to be dependent on IL-23 (Wallace et al., 2014). Selective inhibition of IL-23, demonstrated by drugs like risankizumab and mirikizumab, appears to be effective in the treatment of CD, with emerging evidence suggesting efficacy in UC as well (Misselwitz et al., 2020). Mirikizumab, as a monoclonal antibody, displayed typical pharmacokinetic characteristics, where clearance was observed to increase with body weight and decrease with albumin levels. Additionally, bioavailability was found to decrease with body mass index. This may suggest that administration of mirikizumab treatment and, at the same time, nutritional intervention to stop malnutrition in patients with IBD may be a beneficial combination (Chua et al., 2023).

The characteristics of the analyzed studies that further evaluated the efficacy and safety of novel therapeutic agents approved for IBD treatment are presented in Table 1.

**TABLE 1:** Characteristics and outcomes of clinical studies regarding novel pharmacotherapy in IBD.

Name	Class	Study	Number of patients included in the study	Study Outcomes
Vedolizumab	α4β7 integrin antagonist	VISIBLE 1	216	Patients achieved clinical remission at week 52 • in the subcutaneous vedolizumab group: 46,2% • in the intravenous vedolizumab group: 42,6% • placebo group: 14,3% (Sandborn et al., 2020)
		GEMINI LTS	2,243	Long-term treatment with vedolizumab consistently upheld or enhanced health-related quality of life (HRQOL) in individuals diagnosed with moderate to severely active UC or CD (Loftus et al., 2020)
		VARSITY	769	• superior histological remission compared to adalimumab at week 14 • higher rates of mucosal healing compared to adalimumab at week 52 (Peyrin-Biroulet et al., 2021)
		Study conducted by Onali et al	470	Higher rates of clinical remission in patients treated with vedolizumab compared to ustekinumab (55.5% vs. 42.5%) (Onali et al., 2022)
		VISIBLE 2	644	Clinical remission by week 52 • 48.0% of patients receiving subcutaneous vedolizumab • 34.3% of patients receiving placebo Improved clinical response at week 52 • 52.0% of patients receiving subcutaneous vedolizumab • 44.8% of patiens receiving placebo (Vermeire et al., 2022)
Ustekinumab	monoclonal antibody directed against IL-12 and IL-23	IM-UNITI	397	At week 252 achieved clinical remission • 34.4% of patients receiving ustekinumab treatment every 8 weeks • 28.7% of those receiving treatment every 12 weeks (Sandborn et al., 2022a) Among patients continuing into the long-term treatment extension, remission rates were 54.9% and 45.2% respectively (Sandborn et al., 2022a)
		UNIFI	399	Symptomatic remission at week 152 • 54.1% of pacients receiving ustekinumab every 12 weeks • 56.3% of pacients receiving ustekinumab every 8 weeks (Abreu et al., 2022)
		Multicenter cohort study Dalal et al	108	• 40% of UC patients achieved remission following ustekinumab induction • among patients who underwent dose escalation, over 50% achieved corticosteroid-free remission (Dalal et al., 2022)
Mirikizumab	anti-IL23 p19 monoclonal anibody	LUCENT-1 and LUCENT-2	LUCENT-1: 1,162 LUCENT-2: 544	It showed clinical advantage, also for bowel urgency in individuals with moderately to severely active forms of UC, which comprised 52 weeks of continuous therapy (Dubinsky et al., 2022a)
Guselkumab	anti-IL23 p19 monoclonal anibody	GALAXI-1	309	Superior efficacy versus placebo, and efficacy and safety were similar between guselkumab dose groups at week 12 in individuals with either moderately to severely active CD (Sandborn et al., 2022b)
		QUASAR Phase 2b Induction Study	313	Similar results with the ones obtained from GALAXI-1 study (Peyrin-Biroulet et al., 2023)

**FIGURE 2:**
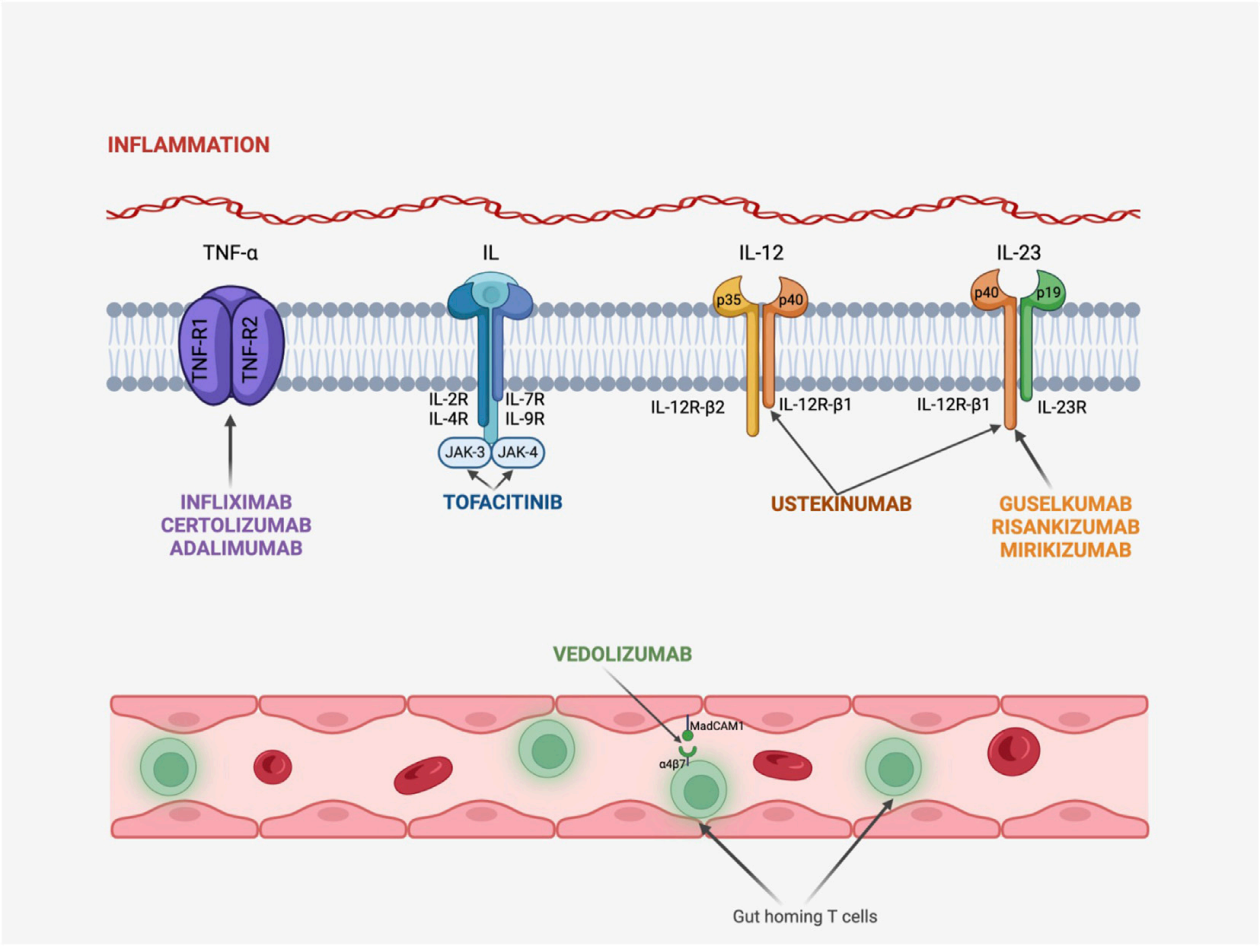
Mechanisms of action for novel therapeutic agents in IBD. (TNF-R, TNF receptor; IL-R, interleukin receptor, JAK, Janus-kinase; Infliximab, Certolizumab and Adalimumab act by binding to TNF-R and blocking TNF-α activity; Tofacitinib binds to several IL-receptors and inhibits JAK signalization; Ustekinumab is capable of binding to p40 fraction from both IL-12 and IL-23, while Guselkumab, Risankizumab and Mirikizumab bind only to IL-23, exhibiting an anti-inflammatory activity; Vedolizumab targets the α4β7 integrin and migrates through the intestine by using T lymphocytes and a specific adhesion molecule called MadCAM-1.)

Nevertheless, in Figure 2 are summarized the mechanisms of action for these drugs.

## 6 Vaccination and IBD

Chronic IBD requires immunosuppressive treatments that further expose individuals to a higher risk of complications and infections (Launay et al., 2015). Live-attenuated vaccines are contraindicated after therapy has been initiated in IBD individuals, but world-wide guidelines recommend various non-live vaccines in order to limit impaired immune responses (Garcillán et al., 2022). However, data is not sufficient regarding the effectiveness of various vaccinations schemes in patients diagnosed with IBD. Nevertheless, the immunological pathways activated through vaccination could negatively influence the evolution of IBD (Launay et al., 2015), as immunosuppressive agents (such as corticosteroids, azathioprine, 6-mercaptopurine, tacrolimus, cyclosporine, adalimumab, golimumab, infliximab, ustekinumab, tofacitinib) could cause difficulty in vaccines to enhance the antibody titre (Shirai et al., 2018). Even more, combination of immunosuppressive therapies increases the prevalence of opportunistic infections in individuals diagnosed with IBD (Ishige et al., 2023). But the mechanisms behind impairment of the immune response are still poorly understood.

Vaccine response is based on both humoral and cellular immunity, as antigens are presented by MHC class II molecules to B and T-lymphocytes through professional antigen-presenting cells (dendritic cells, macrophages), after initiation of phagocytosis (Garcillán et al., 2022; Jiskoot et al., 2019). Several immune complexes can activate cytolytic complement pathway and phagocytic cells can express receptors that will bind to activated complement factors and will stimulate phagocytosis (Jiskoot et al., 2019). The antigen recognition and interaction of dendritic cells and CD4^+^ helper T-cells (surface molecules) activate T helper cells as a co-stimulation mechanism (Jiskoot et al., 2019; Abbas et al., 2014; Delves et al., 2017) and, further, ensure pro-inflammatory cytokine production (Jiskoot et al., 2019; Abbas et al., 2014; Delves et al., 2017).

T cells and cytokines secreted by Th cells (CD4^+^ helper T pathway) therefore mediate elimination or death of infected cells, by activating macrophages through Th1, B cells through Th2 and cytotoxic T cells (Jiskoot et al., 2019; Abbas et al., 2014; Delves et al., 2017). Cytotoxic T lymphocytes (CTLS) are differentiated through CD8^+^ cytotoxic T cells pathway and are directly involved in recognition of foreign antigens (that are expressed on cell surface by MHC class I molecules), production of cytolytic proteins (perforins and granzymes), and subsequently, apoptosis (Jiskoot et al., 2019; Abbas et al., 2014; Delves et al., 2017). This mechanism is also supported by macrophages responsible of killing intracellular pathogens. Cytokines, represented by interleukin-12 and type 1 interferon, promote the ability of CTLS to kill target cells, therefore they are essential for CTLS development (Jiskoot et al., 2019; Abbas et al., 2014; Delves et al., 2017).

On the other hand, antigen binding and Th2 cells production also initiates clonal expansion and antigen specific B-cells differentiation, leading to antibodies production (Jiskoot et al., 2019; Abbas et al., 2014; Delves et al., 2017). Long-lasting plasma cells are therefore differentiated in lymphoid follicles from B cells and can further ensure an anamnestic immune pathway in case of re-exposure to the antigen (Slifka and Amanna, 2019; Zimmermann and Curtis, 2019).

Vaccines are able to exploit immunological adaptative response (Jiskoot et al., 2019; Abbas et al., 2014; Delves et al., 2017). Various Th cells are dependent on the functionality and production of IL-6, IL-23, IL-17 and IL-22, which further have been linked to IBD development (Jiskoot et al., 2019; Abbas et al., 2014; Delves et al., 2017). IBD itself could also alter the effectiveness of vaccination (Jones et al., 2021).

Yet, IBD is not considered a contraindication for inactivated vaccines (*Haemophilus* influenzae type b, recombinant Herpes-Zoster vaccine, diphtheria-tetanus, diphtheria-pertussis-tetanus, hepatitis B vaccine, injectable influenza vaccine, pneumococcal 13- and 23-valent vaccines, Human Papilloma Virus, COVID-19), according to worldwide recommendations, such as those of Centre for Disease Control and Prevention from the USA, The Japanese Society of Gastroenterology, Canada’s National Advisory Committee on Immunization recommendations, British Society of Gastroenterology (Lamb et al., 2019; Jones et al., 2021). Pneumococcal vaccine and annual injectable influenza vaccine are strongly recommended in IBD patients on immunosuppressant treatment, although they are preferred prior to therapy initiation (Lamb et al., 2019). It is generally accepted that non-live vaccines are considered beneficial for individuals (both adults and children) diagnosed with IBD, with no regard to their immune status (Ishige et al., 2023).

On the other hand, live vaccines (measles, mumps, rubella vaccine, polio, varicella zoster vaccine, rotavirus, smallpox, chickenpox, BCG vaccine, yellow fever, oral typhoid Ty21a, oral or nasal influenza vaccine) are not usually administered or recommended for IBD individuals who are receiving immunosuppressive therapy, as future research is necessary in order to assess risk-benefit report (Benchimol et al., 2021). Moreover, live vaccines need to be avoided even after discontinuing immunosuppressive therapy, for at least 3 months (Lamb et al., 2019; Ishige et al., 2023). Intra-uterine infants exposed to biologics have a contraindication in receiving live vaccines for 6 months after birth (Lamb et al., 2019).

The most frequently discussed vaccinations are the ones against influenza virus and pneumococcal infections (Garcillán et al., 2022). A phase III prospective multicentre study evaluated both the efficacy and the 2-year impact safety of anti-H1N1 vaccine in 255 adults diagnosed with IBD (Launay et al., 2015). Although the influenza vaccine provided high rates of immune protection, the serological protection was diminished in patients receiving anti-TNF agents (Launay et al., 2015; Marín et al., 2015). Shirai et al. concluded in their research that it is difficult to obtain immunogenicity, even after vaccination with a quadrivalent inactivated influenza vaccine in individuals treated with infliximab (Shirai et al., 2018). However, a recent randomized clinical trial, conducted by Caldera et al., proved that higher doses of influenza vaccine in IBD individuals who received treatment with anti-TNF agents in monotherapy involved a greater antibody titre compared to standard doses (Caldera et al., 2020). No increase in antibody activity was noted in a prospective clinical trial that included patients with various regimens and influenza vaccine, although the research correlated the absence of immune response with previous annual vaccination that contributed to a seropositive state of all patients (Harrington et al., 2020). A decrease in serum levels of IL-2 has been linked with diminished immune response after influenza vaccination in IBD patients (Marín et al., 2015). However, the ability of B lymphocytes to generate antibodies was amplified after repeated influenza vaccination (Frasca et al., 2016).

Another randomized clinical trial concluded that individuals who were vaccinated against pneumonia using either the 23-valent pneumococcal polysaccharide vaccine or the 13-valent pneumococcal conjugated vaccine while administering immunosuppressive TNF antagonists proved an unbalanced antibody response compared to the ones that did not receive therapy (Kantsø et al., 2015). The European Crohn’s and Colitis Organisation underlines the importance of knowing the antibody titre of individuals diagnosed with IBD, even in case of receiving routine vaccines, such as pneumococcal or hepatitis (Marín et al., 2015). In case of pneumococcal 23-valent vaccine, the polysaccharides are antigens that do not depend on T-lymphocytes activity, but they can be targeted by B-cells that will further produce antibodies against bacteria (Marín et al., 2015). IBD patients, even without receiving treatment, are deficient in memory B cells compared to healthy individuals, due to impaired spleen functionality (Marín et al., 2015), or have B cells’ functionality switched from the naïve B-cell mechanisms (Fleming et al., 2022). Despite all these aberrant immune responses in IBD individuals, another open-label multicentre phase IV clinical trial conducted by Pittet et al. also supported the use of pneumococcal vaccine in those patients, after including more than 300 adults who received 13-valent pneumococcal conjugated vaccine (Pittet et al., 2019).

A prospective and clinically controlled trial conducted by Harrington and colleagues, in which 160 vaccinations with either influenza, pneumococcal or hepatitis B vaccines where administered, included patients grouped in four categories based on the medication regimen (Harrington et al., 2020). Their conclusion supported vaccination in patients with IBD and underlined that vedolizumab treatment led to non-inferior immunogenic response in case of hepatitis B vaccination, when compared to control groups that did not receive immunosuppressive therapy (Harrington et al., 2020). Vedolizumab is not considered by some researchers an immunosuppressive agent, as it provides gut-selectivity (Ishige et al., 2023; Harrington et al., 2020). The ability of hepatitis B vaccine to induce a proper immune response depends on the T lymphocytes response to the antigen, implicitly on the functionality of antigen-presenting cells (Marín et al., 2015).

However, one of the most recently researched vaccine in IBD patients has been SARS-COV-2 vaccine based on mRNA. T-lymphocytes represent a critical pathway of the immune response in vaccination and IBD, as T cells are able to specifically recognise the antigen through MHC class I and II molecules and receptors (Xu et al., 2022). Anti-TNF agents and corticosteroids have been linked with lower serum levels of antibodies post-SARS-COV-2 vaccination in individuals diagnosed with IBD (Kappelman et al., 2021). Another prospective research that included 521 subjects, diagnosed with IBD, concluded that SARS-COV-2 vaccine was successfully received and that those with T-cells deficiencies could benefit of enhanced booster protocols (Xu et al., 2022). Lin et al. also noted that higher values of antibody were found in IBD adults presenting antecedents of COVID-19 infection prior to vaccination, independent on the specific immunosuppressive therapy (Lin et al., 2022). Moreover, infliximab treatment was linked to a diminished and less durable immune activity after administering two doses of vaccine in comparison with vedolizumab (Lin et al., 2022). Another agent that was not involved in attenuated immune responses post-vaccination is ustekinumab (Bordalo Ferreira et al., 2023). Researchers, therefore, recommend SARS-COV-2 vaccination in all patients diagnosed with IBD and receiving anti-TNF agents (Lin et al., 2022), although diminished humoral responses have been noted in those specific populations (Bordalo Ferreira et al., 2023).

Although the precise cause of IBD is under research, the innate and adaptative immune responses appear dysregulated, even before initiation of therapy (Geremia et al., 2014). Production of excessive antimicrobial peptides, stimulation of macrophages and dendritic cells, excessive differentiation of Th1, Th2 and Th17, and increased autophagic mechanisms are seen as aberrant innate immune pathways in IBD, that further lead to chronic and dramatic inflammation (Geremia et al., 2014; Lu et al., 2022). Perturbance between maintaining the beneficial and protective bacterial antigens and removing the intestinal pathogenic bacteria is directly involved in IBD pathogenesis (Geremia et al., 2014; Chen et al., 2021). Intracellular endoplasmic reticulum stress response and autophagic dysfunctionality, dysregulation of Th1 and Th2 mediated responses, chronic inflammatory responses induced by IL-23, expansion of Th17 cells (derived from TGF-beta and IL-6) and epithelial TNF-induced apoptosis are several pathways involved in IBD (Geremia et al., 2014; Larabi et al., 2020). Moreover, IL-12 initiates differentiation of naïve cells (such as CD4^+^ T cells that are able to form Th1 cells). These activated cells are further responsible of IFN-γ production and also ensure the proliferation of NK cells and CTLS, while IL-23 increases inflammatory Th17 responses (Lu et al., 2022). Proper autophagy mechanisms are not only essential to avoid IBD, but also to ensure an optimal response to anti-TNF agents (Larabi et al., 2020). These are some of the reasons why IBD itself could disrupt further immune responses induced by vaccination, as elevated levels of cells expressing IgG after B lymphocytes dysregulation (also known as switching mechanism of B cells) have been noted in case of intestinal inflammation (Fleming et al., 2022). Nevertheless, B-cell depleting agents, molecules that act as TNF blockers and CTLA-4 fusion protein (abatacept) have been associated with the greatest reduction in immunological response after vaccination (Garcillán et al., 2022; Jiskoot et al., 2019). Anti-integrin α4β7, anti-IL-6, anti-IL-12/23, anti-BAFF or anti-IL-17 agents have not been involved in attenuation of vaccine responses (Garcillán et al., 2022; Slifka and Amanna, 2019; Zimmermann and Curtis, 2019). A classification regarding the involvement of IBD treatments in post-vaccination immune responses is presented in Table 2.

**TABLE 2:** IBD therapies and immune responses after several vaccines.

Associated with impaired vaccines immunogenicity	Do not appear to compromise the immune response after several vaccination regimens
• B cell-depleting therapies (rituximab) (Jiskoot et al., 2019; Pittet et al., 2019) • TNF-inhibitors (Launay et al., 2015; Shirai et al., 2018) • cytotoxic T-lymphocyte-associated antigen-4 (CTLA-4) fusion protein (abatacept) (Garcillán et al., 2022; Jiskoot et al., 2019) • corticosteroids (Launay et al., 2015) • thiopurines (Jiskoot et al., 2019) • methotrexate (Jiskoot et al., 2019)	• IL-12/23 inhibitor (ustekinumab) (Bordalo Ferreira et al., 2023; Geremia et al., 2014) • Anti-integrins (vedolizumab) (Bordalo Ferreira et al., 2023; Geremia et al., 2014) • integrin, IL-6, IL-12/23, IL-17, and B-cell activating factor (BAFF) inhibitors (Garcillán et al., 2022; Slifka and Amanna, 2019; Zimmermann and Curtis, 2019)

## 7 General recommendations for patients diagnosed with IBD

Considering the Western diet is a major risk factor for IBD, and with the adaptation to western food, the incidence of IBD is also increasing in African and Asian countries. Therefore the interplay between Western diet and IBD should be extensively studied in clinical trials. Multidisciplinary approach should be included within these studies among individuals with UD or CD, as both appropriate vaccination and diet could influence treatment outcomes. In order to support this approach, recommendations regarding nutritional aspects for patients and healthcare professionals are summarized in Table 3 (UCSF, 2024; Magro et al., 2024).

**TABLE 3:** Dietary recommendations for IBD patients.

Food groups	Recommended during flare-ups	To avoid during flare-ups
Vegetables	- the easiest to digest (asparagus, potatoes) - cooked/mashed/without skin - the juice left after boiling can be added to pasta/rice	- hard-shelled ones - the ones that cause bloating (broccoli, cabbage, cauliflower, lettuce)
Fruits	- applesauce, melon, avocado, bananas, papaya - thermally prepared/peeled/mashed/compote	- fruits with a high fiber content (oranges, dehydrated fruits) - fruits with seeds (strawberries, raspberries)
Cereals	- refined cereals - porridge, potatoes, rice, pasta	- cereals with nuts and seeds - popcorn
Proteins	- lean meat (fish, chicken, turkey), salmon - eggs, tofu - peanut butter/almonds/cashew butter/almond milk	- fatty, fried or hyper-processed meat - whole walnuts
Beverages	- water	- carbonated drinks, coffee, alcohol

IBD patients are recommended to avoid ultra-processed food and to have smaller, more frequent meals that are better tolerated and can maximize nutritional intake (as discussed in Table 3). Additionally, they should consider taking nutritional supplements if solid foods are not well tolerated or appetite is diminished (UCSF, 2024; Magro et al., 2024).

## 8 Conclusion

Biological therapies represent a large area of research regarding autoimmune diseases.

The unknown causes of downgrading of IBD, the diversity of responses to drug therapies, the complexity of case management determine the particularity of each patient. Thus, the continuous study of the evolution of biological therapies and their approach together with the nutritional and psychological aspects remains a challenge for multidisciplinary teams.

In IBD patients, data regarding the effectiveness of various vaccinations schemes is insufficient. Future large, well-designed studies are needed for a more profound insight of the pathogenic mechanisms and novel drugs modes of action with the aim of improving patient identification and pharmacotherapeutic stratification.

## References

[B1] Abbas A. K. Lichtman A. H. Pillai S. (2014). Basic immunology: functions and disorders of the immune system. 4th ed. Philadelphia: Elsevier/Saunders.

[B2] Abreu M. T. Rowbotham D. S. Danese S. Sandborn W. J. Miao Y. Zhang H. (2022). Efficacy and safety of maintenance ustekinumab for ulcerative colitis through 3 Years: UNIFI long-term extension. J. Crohns Colitis 16 (8), 1222–1234. 10.1093/ecco-jcc/jjac030 35239968 PMC9426670

[B3] Akram W. Garud N. Joshi R. (2019). Role of inulin as prebiotics on inflammatory bowel disease. Ther 13, 1–8. 10.5582/ddt.2019.01000 30880316

[B4] Al-Bawardy B. Shivashankar R. Proctor D. D. (2021). Novel and emerging therapies for inflammatory bowel disease. Front. Pharmacol. 12, 651415. 10.3389/fphar.2021.651415 33935763 PMC8080036

[B5] Baeten D. Østergaard M. Wei J. C. Sieper J. Järvinen P. Tam L. S. (2018). Risankizumab, an IL-23 inhibitor, for ankylosing spondylitis: results of a randomised, double-blind, placebo-controlled, proof-of-concept, dose-finding phase 2 study. Ann. Rheum. Dis. 77 (9), 1295–1302. 10.1136/annrheumdis-2018-213328 29945918 PMC6104676

[B6] Benchimol E. I. Tse F. Carroll M. W. deBruyn J. C. McNeil S. A. Pham-Huy A. (2021). Canadian association of Gastroenterology clinical practice guideline for immunizations in patients with inflammatory bowel disease (IBD)-Part 1: live vaccines. Gastroenterology 161 (2), 669–680.e0. 10.1053/j.gastro.2020.12.079 33617891

[B7] Benson J. M. Peritt D. Scallon B. J. Heavner G. A. Shealy D. J. Giles-Komar J. M. (2011). Discovery and mechanism of ustekinumab: a human monoclonal antibody targeting interleukin-12 and interleukin-23 for treatment of immune-mediated disorders. MAbs 3 (6), 535–545. 10.4161/mabs.3.6.17815 22123062 PMC3242840

[B8] Bhattacharya S. Cross R. K. (2020). An overview of novel and emerging therapies for inflammatory bowel disease EMJ gastroenterol. 9(1):91–101. 10.33590/emjgastroenterol/20-00166

[B9] Bordalo Ferreira F. Rafael M. A. Coimbra L. Boavida N. Arrobas F. Pereira C. F. (2023). Anti-tumor necrosis factor therapy is associated with attenuated humoral response to SARS-COV-2 vaccines in patients with inflammatory bowel disease. Vaccine 41 (26), 3862–3871. 10.1016/j.vaccine.2023.05.012 37202269 PMC10165058

[B10] Bretto E. Ribaldone D. G. Caviglia G. P. Saracco G. M. Bugianesi E. Frara S. (2023). Inflammatory bowel disease: emerging therapies and future treatment strategies. Biomedicines 11 (8), 2249. 10.3390/biomedicines11082249 37626745 PMC10452708

[B11] Briskin M. Winsor-Hines D. Shyjan A. Cochran N. Bloom S. Wilson J. (1997). Human mucosal addressin cell adhesion molecule-1 is preferentially expressed in intestinal tract and associated lymphoid tissue. Am. J. Pathol. 151 (1), 97–110. 9212736 PMC1857942

[B12] Brück J. Holstein J. Glocova I. Seidel U. Geisel J. Kanno T. (2017). Nutritional control of IL-23/Th17-mediated autoimmune disease through HO-1/STAT3 activation. Sci. Rep. 7, 44482. 10.1038/srep44482 28290522 PMC5349589

[B13] Caldera F. Hillman L. Saha S. Wald A. Grimes I. Zhang Y. (2020). Immunogenicity of high dose influenza vaccine for patients with inflammatory bowel disease on anti-TNF monotherapy: a randomized clinical trial. Inflamm. Bowel Dis. 26 (4), 593–602. 10.1093/ibd/izz164 31504526

[B14] Campmans-Kuijpers M. J. E. Dijkstra G. (2021). Food and food groups in inflammatory bowel disease (IBD): the design of the groningen anti-inflammatory diet (GrAID). Nutrients 13 (4), 1067. 10.3390/nu13041067 33806061 PMC8064481

[B15] Centers for Disease Control and Prevention (2023). Data and statistics. Available at: https://www.cdc.gov/ibd/data-and-statistics/prevalence.html (Accessed November 26, 2023).

[B16] Cheifetz A. S. Gianotti R. Luber R. Gibson P. R. (2017). Complementary and alternative Medicines used by patients with inflammatory bowel diseases. Gastroenterology 152, 415–429. 10.1053/j.gastro.2016.10.004 27743873

[B17] Chen Y. Cui W. Li X. Yang H. (2021). Interaction between commensal bacteria, immune response and the intestinal barrier in inflammatory bowel disease. Front. Immunol. 12, 761981. 10.3389/fimmu.2021.761981 34858414 PMC8632219

[B18] Chiba M. Abe T. Tsuda H. Sugawara T. Tsuda S. Tozawa H. (2010). Lifestyle-related disease in Crohn’s disease: relapse prevention by a semi-vegetarian diet. World J. Gastroenterol. 16, 2484–2495. 10.3748/wjg.v16.i20.2484 20503448 PMC2877178

[B19] Chua L. Friedrich S. Zhang X. C. (2023). Mirikizumab pharmacokinetics in patients with moderately to severely active ulcerative colitis: results from phase III LUCENT studies. Clin. Pharmacokinet. 62 (10), 1479–1491. 10.1007/s40262-023-01281-z 37610533 PMC10520102

[B20] Clement B. De Felice K. Afzali A. (2023). Indications and safety of newer IBD treatments in the older patient. Curr. Gastroenterol. Rep. 25 (7), 160–168. 10.1007/s11894-023-00874-9 37227615 PMC10209934

[B21] Dalal R. S. Esckilsen S. Barnes E. L. Pruce J. C. Marcus J. Allegretti J. R. (2022). Predictors and outcomes of ustekinumab dose intensification in ulcerative colitis: a multicenter cohort study. Clin. Gastroenterol. Hepatol. 20 (10), 2399–2401.e4. 10.1016/j.cgh.2021.03.028 33775893 PMC8464615

[B22] Delves P. J. Martin S. J. Burton D. R. Roitt I. M. (2017). Roitt’s essential immunology. 13th edn. Hoboken: Wiley.

[B23] Dubinsky M. Ma C. Griffith J. Crowell M. Neimark E. Kligys K. (2023a). Matching-adjusted indirect comparison between risankizumab and ustekinumab for induction and maintenance treatment of moderately to severely active crohn's disease. Adv. Ther. 40 (9), 3896–3911. 10.1007/s12325-023-02546-6 37368103 PMC10427520

[B24] Dubinsky M. C. Clemow D. B. Hunter G. T. Li X. Vermeire S. Hisamatsu T. (2022a). Clinical effect of mirikizumab treatment on bowel urgency in patients with moderately to severely active ulcerative colitis and the clinical relevance of bowel urgency improvement for disease remission. Crohns Colitis 360 (1), otac044. 10.1093/crocol/otac044 PMC9802448 36777368

[B25] Dubinsky M. C. Jairath V. Feagan B. G. Naegeli A. N. Tuttle J. Morris N. (2023b). Changes in health-related quality of life and associations with improvements in clinical efficacy: a Phase 2 study of mirikizumab in patients with ulcerative colitis. BMJ Open Gastroenterol. 10 (1), e001115. 10.1136/bmjgast-2023-001115 PMC10069555 37001911

[B26] Dubinsky M. C. Panaccione R. Lewis J. D. Sands B. E. Hibi T. Lee S. D. (2022b). Impact of bowel urgency on quality of life and clinical outcomes in patients with ulcerative colitis. Crohns Colitis 360 (3), otac016. 10.1093/crocol/otac016 PMC9802402 36777426

[B27] Elhag D. A. Kumar M. Saadaoui M. Akobeng A. K. Al-Mudahka F. Elawad M. (2022). Inflammatory bowel disease treatments and predictive biomarkers of therapeutic response. Int. J. Mol. Sci. 23 (13), 6966. 10.3390/ijms23136966 35805965 PMC9266456

[B28] European Medicines Agency. (2023a). Product information. Available at: https://www.ema.europa.eu/en/documents/product-information/skyrizi-epar-product-information_en.pdf (Accessed October 12, 2023).

[B98] European Medicines Agency. (2023b). Product information. Available at: https://www.ema.europa.eu/en/documents/product-information/omvoh-epar-product-information_en.pdf (Accessed October 12, 2023).

[B29] Feagan B. G. Rutgeerts P. Sands B. E. Hanauer S. Colombel J. F. Sandborn W. J. (2013). Vedolizumab as induction and maintenance therapy for ulcerative colitis. N. Engl. J. Med. 369, 699–710. 10.1056/NEJMoa1215734 23964932

[B30] Feuerstein J. D. Isaacs K. L. Schneider Y. Siddique S. M. Falck-Ytter Y. Singh S. AGA Institute Clinical Guidelines Committee (2020). AGA clinical practice guidelines on the management of moderate to severe ulcerative colitis. Gastroenterology 158 (5), 1450–1461. 10.1053/j.gastro.2020.01.006 31945371 PMC7175923

[B31] Fleming A. Castro-Dopico T. Clatworthy M. R. (2022). B cell class switching in intestinal immunity in health and disease. Scand. J. Immunol. 95 (2), e13139. 10.1111/sji.13139 34978077 PMC9285483

[B32] Frasca D. Diaz A. Romero M. Blomberg B. B. (2016). The generation of memory B cells is maintained, but the antibody response is not, in the elderly after repeated influenza immunizations. Vaccine 34, 2834–2840. 10.1016/j.vaccine.2016.04.023 27108193 PMC4876633

[B33] Fujita Y. Sugaya T. Tanaka T. Tominaga K. Yoshihara S. (2021). Ustekinumab as the first biological agent for crohn's disease in a 10-year-old girl. Tohoku J. Exp. Med. 255 (1), 57–60. 10.1620/tjem.255.57 34588346

[B34] Gao C. Zhou Y. Chen Z. Li H. Xiao Y. Hao W. (2022). Turmeric-derived nanovesicles as novel nanobiologics for targeted therapy of ulcerative colitis. Theranostics 12 (12), 5596–5614. 10.7150/thno.73650 35910802 PMC9330521

[B35] Garcillán B. Salavert M. Regueiro J. R. Díaz-Castroverde S. (2022). Response to vaccines in patients with immune-mediated inflammatory diseases: a narrative review. Vaccines (Basel) 10 (2), 297. 10.3390/vaccines10020297 35214755 PMC8877652

[B36] GBD 2017 Inflammatory Bowel Diseases Collaborators (2020). The global, regional, and national burden of inflammatory bowel disease in 195 countries and territories, 1990–2017: a systematic analysis for the Global Burden of Disease Study 2017. Lancet Gastroenterology Hepatology 5 (1), 17–30. 10.1016/S2468-1253(19)30333-4 31648971 PMC7026709

[B37] Geremia A. Biancheri P. Allan P. Corazza G. R. Di Sabatino A. (2014). Innate and adaptive immunity in inflammatory bowel disease. Autoimmun. Rev. 13 (1), 3–10. 10.1016/j.autrev.2013.06.004 23774107

[B38] Godala M. Gaszynska E. Zatorski H. Małecka-Wojciesko E. (2022). Dietary interventions in inflammatory bowel disease. Nutrients 14, 4261. 10.3390/nu14204261 36296945 PMC9607252

[B39] Gottlieb Z. S. Sands B. E. (2022). Personalised medicine with IL-23 blockers: myth or reality? J. Crohns Colitis 16 (Suppl. ment_2), ii73–ii94. 10.1093/ecco-jcc/jjab190 35553661 PMC9113162

[B40] Hanžel J. D'Haens G. R. (2020). Anti-interleukin-23 agents for the treatment of ulcerative colitis. Expert Opin. Biol. Ther. 20 (4), 399–406. 10.1080/14712598.2020.1697227 31760827

[B41] Harrington J. E. Hamilton R. E. Ganley-Leal L. Farraye F. A. Wasan S. K. (2020). The immunogenicity of the influenza, pneumococcal, and hepatitis B vaccines in patients with inflammatory bowel disease treated with vedolizumab. Crohns Colitis 360 (4), otaa082. 10.1093/crocol/otaa082 PMC9802260 36777751

[B42] Horst S. Cross R. K. (2023). Clinical evaluation of risankizumab in the treatment of adults with moderately to severely active crohn's disease: patient selection and reported outcomes. Drug Des. Devel Ther. 17, 273–282. 10.2147/DDDT.S379446 PMC9899013 36747585

[B43] Ishige T. Shimizu T. Watanabe K. Arai K. Kamei K. Kudo T. (2023). Expert consensus on vaccination in patients with inflammatory bowel disease in Japan. J. Gastroenterol. 58 (2), 135–157. 10.1007/s00535-022-01953-w 36629948 PMC9838549

[B44] Jain A. Nguyen N. H. Proudfoot J. A. Martin C. F. Sandborn W. J. Kappelman M. D. (2019). Impact of obesity on disease activity and patient-reported outcomes measurement information System (PROMIS) in inflammatory bowel diseases. Am. J. Gastroenterol. 114 (4), 630–639. 10.14309/ajg.0000000000000197 30865012 PMC6824268

[B45] Jiskoot W. Kersten G. F. A. Mastrobattista E. Slütter B. (2019). Vaccines. Pharm. Biotechnol. 14, 281–304. 10.1007/978-3-030-00710-2_14

[B46] Jones J. L. Tse F. Carroll M. W. deBruyn J. C. McNeil S. A. Pham-Huy A. (2021). Canadian association of Gastroenterology clinical practice guideline for immunizations in patients with inflammatory bowel disease (IBD)-Part 2: inactivated vaccines. Gastroenterology 161 (2), 681–700. 10.1053/j.gastro.2021.04.034 34334167

[B47] Kantsø B. Halkjær S. I. Thomsen O. Ø. Belard E. Gottschalck I. B. Jørgensen C. S. (2015). Immunosuppressive drugs impairs antibody response of the polysaccharide and conjugated pneumococcal vaccines in patients with Crohn's disease. Vaccine 33 (41), 5464–5469. 10.1016/j.vaccine.2015.08.011 26275480

[B48] Kappelman M. D. Weaver K. N. Zhang X. Dai X. Watkins R. Adler J. (2021). Factors affecting initial humoral immune response to SARS-cov-2 vaccines among patients with inflammatory bowel diseases. Am. J. Gastroenterol. 117 (3), 462–469. 10.14309/ajg.0000000000001619 35029167

[B49] Keam S. J. (2023). Mirikizumab: first approval. Drugs 83 (11), 1045–1052. 10.1007/s40265-023-01909-1 37389706

[B50] Lamb C. A. Kennedy N. A. Raine T. Hendy P. A. Smith P. J. Limdi J. K. (2019). British Society of Gastroenterology consensus guidelines on the management of inflammatory bowel disease in adults. Gut 68 (Suppl. 3), s1–s106. 10.1136/gutjnl-2019-318484 31562236 PMC6872448

[B51] Larabi A. Barnich N. Nguyen H. T. T. (2020). New insights into the interplay between autophagy, gut microbiota and inflammatory responses in IBD. Autophagy 16 (1), 38–51. 10.1080/15548627.2019.1635384 31286804 PMC6984609

[B52] Launay O. Abitbol V. Krivine A. Slama L. B. Bourreille A. Dupas J. L. (2015). Immunogenicity and safety of influenza vaccine in inflammatory bowel disease patients treated or not with immunomodulators and/or biologics: a two-year prospective study. J. Crohns Colitis 9 (12), 1096–1107. 10.1093/ecco-jcc/jjv152 26351392

[B53] Lin S. Kennedy N. A. Saifuddin A. Sandoval D. M. Reynolds C. J. Seoane R. C. (2022). Antibody decay, T cell immunity and breakthrough infections following two SARS-CoV-2 vaccine doses in inflammatory bowel disease patients treated with infliximab and vedolizumab. Nat. Commun. 13 (1), 1379. 10.1038/s41467-022-28517-z 35296643 PMC8927425

[B54] Loftus E. V. Jr Colombel J. F. Feagan B. G. Vermeire S. Sandborn W. J. Sands B. E. (2017). Long-term efficacy of vedolizumab for ulcerative colitis. J. Crohns Colitis 11 (4), 400–411. 10.1093/ecco-jcc/jjw177 27683800

[B55] Loftus E. V. Jr Feagan B. G. Panaccione R. Colombel J. F. Sandborn W. J. Sands B. E. (2020). Long-term safety of vedolizumab for inflammatory bowel disease. Aliment. Pharmacol. Ther. 52 (8), 1353–1365. 10.1111/apt.16060 32876349 PMC7540482

[B56] Lu Q. Yang M. F. Liang Y. J. Xu J. Xu H. M. Nie Y. Q. (2022). Immunology of inflammatory bowel disease: molecular mechanisms and therapeutics. J. Inflamm. Res. 15, 1825–1844. 10.2147/JIR.S353038 35310454 PMC8928114

[B57] Luzentales-Simpson M. Pang Y. C. F. Zhang A. Sousa J. A. Sly L. M. (2021). Vedolizumab: potential mechanisms of action for reducing pathological inflammation in inflammatory bowel diseases. Front. Cell Dev. Biol. 9, 612830. 10.3389/fcell.2021.612830 33614645 PMC7887288

[B58] Magro D. O. Sassaki L. Y. Chebli J. M. F. (2024). Interaction between diet and genetics in patients with inflammatory bowel disease. World J. Gastroenterol. 30 (12), 1644–1650. 10.3748/wjg.v30.i12.1644 38617734 PMC11008370

[B59] Marín A. C. Gisbert J. P. Chaparro M. (2015). Immunogenicity and mechanisms impairing the response to vaccines in inflammatory bowel disease. World J. Gastroenterol. 21 (40), 11273–11281. 10.3748/wjg.v21.i40.11273 26527572 PMC4616204

[B60] Misselwitz B. Juillerat P. Sulz M. C. Siegmund B. Brand S. Ibdnet S. an official working group of the Swiss Society of Gastroenterology (2020). Emerging treatment options in inflammatory bowel disease: Janus kinases, stem cells, and more. Digestion 101 (Suppl. 1), 69–82. 10.1159/000507782 32570252

[B61] Narula N. Wong E. C. L. Dehghan M. Mente A. Rangarajan S. Lanas F. (2021). Association of ultra‐processed food intake with risk of inflammatory bowel disease: prospective cohort study. BMJ 374, n1554. 10.1136/bmj.n1554 34261638 PMC8279036

[B62] Nguyen N. H. Ohno-Machado L. Sandborn W. J. Singh S. (2019). Obesity is independently associated with higher annual burden and costs of hospitalization in patients with inflammatory bowel diseases. Clin. Gastroenterol. Hepatol. 17 (4), 709–718. 10.1016/j.cgh.2018.07.004 30012429

[B63] Noviello D. Mager R. Roda G. Borroni R. G. Fiorino G. Vetrano S. (2021). The IL23-IL17 immune Axis in the treatment of ulcerative colitis: successes, defeats, and ongoing challenges. Front. Immunol. 12, 611256. 10.3389/fimmu.2021.611256 34079536 PMC8165319

[B64] Ohmatsu H. Kadono T. Sugaya M. Tomita M. Kai H. Miyagaki T. (2010). α4β7 Integrin is essential for contact hypersensitivity by regulating migration of T cells to skin. J. Allergy Clin. Immunol. 126 (6), 1267–1276. 10.1016/j.jaci.2010.08.048 21047673

[B65] Oligschlaeger Y. Yadati T. Houben T. Condello Oliván C. M. Shiri-Sverdlov R. (2019). Inflammatory bowel disease: a stressed Gut/Feeling. Cells 8 (7), 659. 10.3390/cells8070659 31262067 PMC6678997

[B66] Onali S. Pugliese D. Caprioli F. A. Orlando A. Biancone L. Nardone O. M. (2022). An objective comparison of vedolizumab and ustekinumab effectiveness in crohn's disease patients' failure to TNF-alpha inhibitors. Am. J. Gastroenterol. 117 (8), 1279–1287. 10.14309/ajg.0000000000001773 35467558

[B67] Pang X. He X. Qiu Z. Zhang H. Xie R. Liu Z. (2023). Targeting integrin pathways: mechanisms and advances in therapy. Signal Transduct. Target Ther. 8 (1), 1. 10.1038/s41392-022-01259-6 36588107 PMC9805914

[B68] Parigi T. L. Iacucci M. Ghosh S. (2022). Blockade of IL-23: what is in the pipeline? J. Crohns Colitis 16 (Suppl. ment_2), ii64–ii72. 10.1093/ecco-jcc/jjab185 35553666 PMC9097679

[B69] Peyrin-Biroulet L. Allegretti J. R. Rubin D. T. Bressler B. Germinaro M. Huang K. G. QUASAR Study (2023). Guselkumab in patients with moderately to severely active ulcerative colitis: QUASAR phase 2b induction study. Gastroenterology 165 (23), 1443–1457. S0016-5085. 10.1053/j.gastro.2023.08.038 37659673

[B70] Peyrin-Biroulet L. Loftus E. V. Jr Colombel J. F. Danese S. Rogers R. Bornstein J. D. (2021). Histologic outcomes with vedolizumab versus adalimumab in ulcerative colitis: results from an efficacy and safety study of vedolizumab intravenous compared to adalimumab subcutaneous in participants with ulcerative colitis (VARSITY). Gastroenterology 161 (4), 1156–1167.e3. 10.1053/j.gastro.2021.06.015 34144047

[B71] Pittet L. F. Verolet C. M. Michetti P. Girardin M. Juillerat P. Mottet C. (2019). High immunogenicity of the pneumococcal conjugated vaccine in immunocompromised adults with inflammatory bowel disease. Am. J. Gastroenterol. 114 (7), 1130–1141. 10.14309/ajg.0000000000000289 31205131

[B72] Risankizumab for psoriasis (2020). Risankizumab for psoriasis. Aust. Prescr. 43 (2), 70–71. 10.18773/austprescr.2020.015 32346218 PMC7186275

[B73] Romanian National Health House of Inssurances (2017). Therapeutic protocols. Available at: http://www.casan.ro/cassam/media/pageFiles/LISTA%20PROTOCOALELOR%20TERAPEUTICE%20CU%20MODIFICARILE%20SI%20COMPLETARILE%20ULTERIOARE-%20NOIEMBRIE%202017.pdf (Accessed November 26, 2023).

[B74] Romanian National Health House of Insurrances (2023). List of approved therapeutic protocols by ministerial order. Available at: http://cas.cnas.ro/page/lista-protocoalelor-terapeutice-aprobate-prin-ordinul-ms-cnas-nr-1301-500-2008-cu-modificarile-si-completarile-ulterioare.html (Accessed November 26, 2023).

[B75] Romanian National Health House of Insurrances (2021). List of therapeutic protocols. Available at: http://cas.cnas.ro/media/pageFiles/lista%20protocoalelor%20terapeutice%20mai.4%20%20%202021.pdf (Accessed November 26, 2023).

[B76] Roncoroni L. Gori R. Elli L. Tontini G. E. Doneda L. Norsa L. (2022). Nutrition in patients with inflammatory bowel diseases: a narrative review. Nutrients 14, 751. 10.3390/nu14040751 35215401 PMC8879392

[B77] Sakkas L. I. Zafiriou E. Bogdanos D. P. (2019). Mini review: new treatments in psoriatic arthritis. Focus on the IL-23/17 Axis. Front. Pharmacol. 10, 872. 10.3389/fphar.2019.00872 31447673 PMC6691125

[B78] Sandborn W. J. Baert F. Danese S. Krznarić Ž. Kobayashi T. Yao X. (2020). Efficacy and safety of vedolizumab subcutaneous formulation in a randomized trial of patients with ulcerative colitis. Gastroenterology 158 (3), 562–572. 10.1053/j.gastro.2019.08.027 31470005

[B79] Sandborn W. J. D'Haens G. R. Reinisch W. Panés J. Chan D. Gonzalez S. (2022b). GALAXI-1 investigators. Guselkumab for the treatment of crohn's disease: induction results from the phase 2 GALAXI-1 study. Gastroenterology 162 (6), 1650–1664.e8. 10.1053/j.gastro.2022.01.047 35134323

[B80] Sandborn W. J. Rebuck R. Wang Y. Zou B. Adedokun O. J. Gasink C. (2022a). Five-year efficacy and safety of ustekinumab treatment in crohn's disease: the IM-UNITI trial. Clin. Gastroenterol. Hepatol. 20 (3), 578–590.e4. 10.1016/j.cgh.2021.02.025 33618023 PMC8374005

[B81] Sandborn W. J. Su C. Sands B. E. D'Haens G. R. Vermeire S. Schreiber S. (2017). Tofacitinib as induction and maintenance therapy for ulcerative colitis. N. Engl. J. Med. 376 (18), 1723–1736. 10.1056/NEJMoa1606910 28467869

[B82] Sarkar A. Lehto S. M. Harty S. Dinan T. G. Cryan J. F. Burnet P. W. J. (2016). Psychobiotics and the manipulation of bacteria-gut-brain signals. Trends Neurosci. 39, 763–781. 10.1016/j.tins.2016.09.002 27793434 PMC5102282

[B83] Shirai S. Hara M. Sakata Y. Tsuruoka N. Yamamoto K. Shimoda R. (2018). Immunogenicity of quadrivalent influenza vaccine for patients with inflammatory bowel disease undergoing immunosuppressive therapy. Inflamm. Bowel Dis. 24 (5), 1082–1091. 10.1093/ibd/izx101 29538682 PMC6176891

[B84] Slifka M. K. Amanna I. J. (2019). Role of multivalency and antigenic threshold in generating protective antibody responses. Front. Immunol. 10, 956. 10.3389/fimmu.2019.00956 31118935 PMC6504826

[B85] Sobolewska-Włodarczyk A. Walecka-Kapica E. Włodarczyk M. Gąsiorowska A. (2023). Nutritional status indicators as a predictor of achieving remission at week 14 during vedolizumab therapy in patients with ulcerative colitis: a pilot study. Nutrients 15 (1), 240. 10.3390/nu15010240 36615897 PMC9824159

[B86] Steere B. Beidler C. Martin A. Bright S. Kikly K. Benschop R. J. (2023). Generation and characterization of mirikizumab, a humanized monoclonal antibody targeting the p19 subunit of IL-23. J. Pharmacol. Exp. Ther. 387, 180–187. JPET-AR-2022-001512. 10.1124/jpet.122.001512 37714687

[B87] Sulz M. C. Burri E. Michetti P. Rogler G. Peyrin-Biroulet L. Seibold F. on behalf of the Swiss IBDnet, an official working group of the Swiss Society of Gastroenterolog, (2020). Treatment algorithms for crohn's disease. Digestion 101 (Suppl. 1), 43–57. 10.1159/000506364 32172251

[B88] Suskind D. L. Wahbeh G. Gregory N. Vendettuoli H. Christie D. (2014). Nutritional therapy in pediatric Crohn disease: the specific carbohydrate diet. J. Pediatr. Gastroenterol. Nutr. 58, 87–91. 10.1097/MPG.0000000000000103 24048168

[B89] Torres J. Ellul P. Langhorst J. Mikocka-Walus A. Barreiro-de Acosta M. Basnayake C. (2019). European Crohn’s and colitis organisation topical review on complementary medicine and psychotherapy in inflammatory bowel disease. J. Crohns Colitis 13, 673–685e. 10.1093/ecco-jcc/jjz051 30820529

[B90] UCSF (2024). Available at: https://www.ucsfhealth.org/education/nutrition-tips-for-inflammatory-bowel-disease (Accessed on June 23, 2024).

[B91] Valenti M. Narcisi A. Pavia G. Gargiulo L. Costanzo A. (2022). What can IBD specialists learn from IL-23 trials in dermatology? J. Crohns Colitis 16 (Suppl. ment_2), ii20–ii29. 10.1093/ecco-jcc/jjac023 35553663 PMC9097670

[B92] Vermeire S. Colombel J.-F. Feagan B. G. Sandborn W. J. Sands B. E. Danese S. (2019). OP26 Long-term safety of vedolizumab in ulcerative colitis and Crohn’s disease: final results from the GEMINI LTS study. J. Crohns Colitis 13 (Suppl. 1), S018–S020. 10.1093/ecco-jcc/jjy222.025

[B93] Vermeire S. D'Haens G. Baert F. Danese S. Kobayashi T. Loftus E. V. (2022). Efficacy and safety of subcutaneous vedolizumab in patients with moderately to severely active crohn's disease: results from the VISIBLE 2 randomised trial. J. Crohns Colitis 16 (1), 27–38. 10.1093/ecco-jcc/jjab133 34402887 PMC8797168

[B94] Vermeire S. Loftus E. V. Jr Colombel J. F. Feagan B. G. Sandborn W. J. Sands B. E. (2017). Long-term efficacy of vedolizumab for crohn's disease. J. Crohns Colitis 11 (4), 412–424. 10.1093/ecco-jcc/jjw176 27683798

[B95] Wallace K. L. Zheng L. B. Kanazawa Y. Shih D. Q. (2014). Immunopathology of inflammatory bowel disease. World J. Gastroenterol. 20 (1), 6–21. 10.3748/wjg.v20.i1.6 24415853 PMC3886033

[B96] Xu A. M. Li D. Ebinger J. E. Mengesha E. Elyanow R. Gittelman R. M. (2022). Differences in SARS-CoV-2 vaccine response dynamics between class-I- and class-II-specific T-cell receptors in inflammatory bowel disease. Front. Immunol. 13, 880190. 10.3389/fimmu.2022.880190 35464463 PMC9024211

[B97] Zimmermann P. Curtis N. (2019). Factors that influence the immune response to vaccination. Clin. Microbiol. Rev. 32(2):e000844–18. 10.1128/CMR.00084-18 PMC6431125 30867162

